# Nanoscale crystal imperfection-induced characterization changes of manganite nanolayers with various crystallographic textures

**DOI:** 10.1186/1556-276X-8-345

**Published:** 2013-08-06

**Authors:** Yuan-Chang Liang, Hua Zhong, Wen-Kai Liao

**Affiliations:** 1 Institute of Materials Engineering, National Taiwan Ocean University, Keelung 20224, Taiwan

**Keywords:** Crystallographic texture, Manganite, Epitaxy, Magnetic properties, Buffering

## Abstract

(La,Sr)MnO_3_ (LSMO) nanolayers with various crystallographic textures were grown on the sapphire substrate with and without In_2_O_3_ epitaxial buffering. The LSMO nanolayer with In_2_O_3_ epitaxial buffering has a (110) preferred orientation. However, the nanolayer without buffering shows a highly (100)-oriented texture. Detailed microstructure analyses show that the LSMO nanolayer with In_2_O_3_ epitaxial buffering has a high degree of nanoscale disordered regions (such as subgrain boundaries and incoherent heterointerfaces) in the film. These structural inhomogeneities caused a low degree of ferromagnetic ordering in LSMO with In_2_O_3_ epitaxial buffering, which leads to a lower saturation magnetization value and Curie temperature, and higher coercivity and resistivity.

## Background

Because of their versatile physical properties, various transition metal oxides, specifically perovskite-based manganites, have attracted considerable scientific and technological attention [1-3]. There is potential for the application of La_1 - *x*_Sr_*x*_MnO_3_ (LSMO) in the magnetic storage device and spin-sensitive device field, or it can be used as an important hole-doping material to construct microelectronic devices [2,4,5]. To realize nanodevice applications with high efficiency, it is imperative that LSMO thin films be fabricated on a nanometric scale.

High-quality epitaxial manganite films with specific orientations are essential for the next-generation of microelectronic and magnetic devices. However, single-crystalline perovskite oxide substrates are expensive, and a large diameter substrate is currently technologically unavailable. These factors hinder the practical application of epitaxial LSMO films in the electronic industry [4,6]. Two factors might cause lattice stress in nanoscale manganite thin films. An ultra-thin LSMO epilayer grown on the lattice-mismatched perovskite oxide substrate usually induces built-in stresses in the film, which greatly affect its physical properties [4,7-9]. Moreover, a large thermal expansion coefficient (TEC) difference between the film and substrate also significantly affects the lattice stress in nanoscale manganite thin films. In comparison to randomly oriented thin films, the highly crystallographic textured film usually exhibits superior crystal quality. If the TEC value of a substrate and film is similar, then highly textured ultra-thin polycrystalline LSMO films would not suffer from the lattice distortion that was caused by a lattice mismatch on the single crystalline substrates. This might be promising for practical applications in devices. The sapphire substrate and LSMO have similar TEC sizes [10]. Sapphire substrates can be fabricated with a large diameter and relatively low cost in comparison to perovskite oxide substrates. Such fabrication could attain the practical mass production of a device. Moreover, to form functional heterostructure microelectronic devices, sapphire substrates can be used to integrate LSMO nanofilms with other high-quality optoelectronic thin films [11,12]. During this project, two different crystallographic textured LSMO thin films with a nanoscale thickness were grown using In_2_O_3_ epitaxial underlayering. These films did not suffer lattice stress. These results enable an analysis of the correlation between nanoscale crystal imperfections and manganite nanofilm physical properties.

## Methods

LSMO nanolayers (the Sr content is approximately 39%) with thickness of approximately 60 nm were grown on the *c*-axis-oriented sapphire substrates with and without 40-nm-thick In_2_O_3_ (222) epitaxial buffering. The deposition of the In_2_O_3_ epitaxy layers and LSMO nanolayers was performed using a radiofrequency magnetron-sputtering system. During the deposition, the substrate temperature for the thin-film growth of the In_2_O_3_ epitaxy and LSMO nanolayer was kept at 600°C and 750°C, respectively. Moreover, the gas pressure of deposition was fixed at 10 mTorr with an Ar/O_2_ ratio of 3:1. The as-synthesized samples are further annealed in air ambient at 950°C for 30 min.

The crystal structure of the samples was investigated by X-ray diffraction (XRD) with Cu K*α* radiation. The detailed microstructure of the as-synthesized samples was characterized by scanning electron microscopy (SEM) and high-resolution transmission electron microscopy (HRTEM). The composition analysis was performed using energy dispersive X-ray spectrometer (EDS) attached to the TEM. The surface morphology of the LSMO nanolayers was investigated by atomic force microscopy (AFM) with an area size of 2 μm × 2 μm. The surface current images of the LSMO nanolayers were also observed using conductive atomic force microscopy (CAFM) with PtIr tips. A superconducting quantum interference device magnetometer was used to measure the magnetic properties of the samples.

## Results and discussion

**Figure 1:**
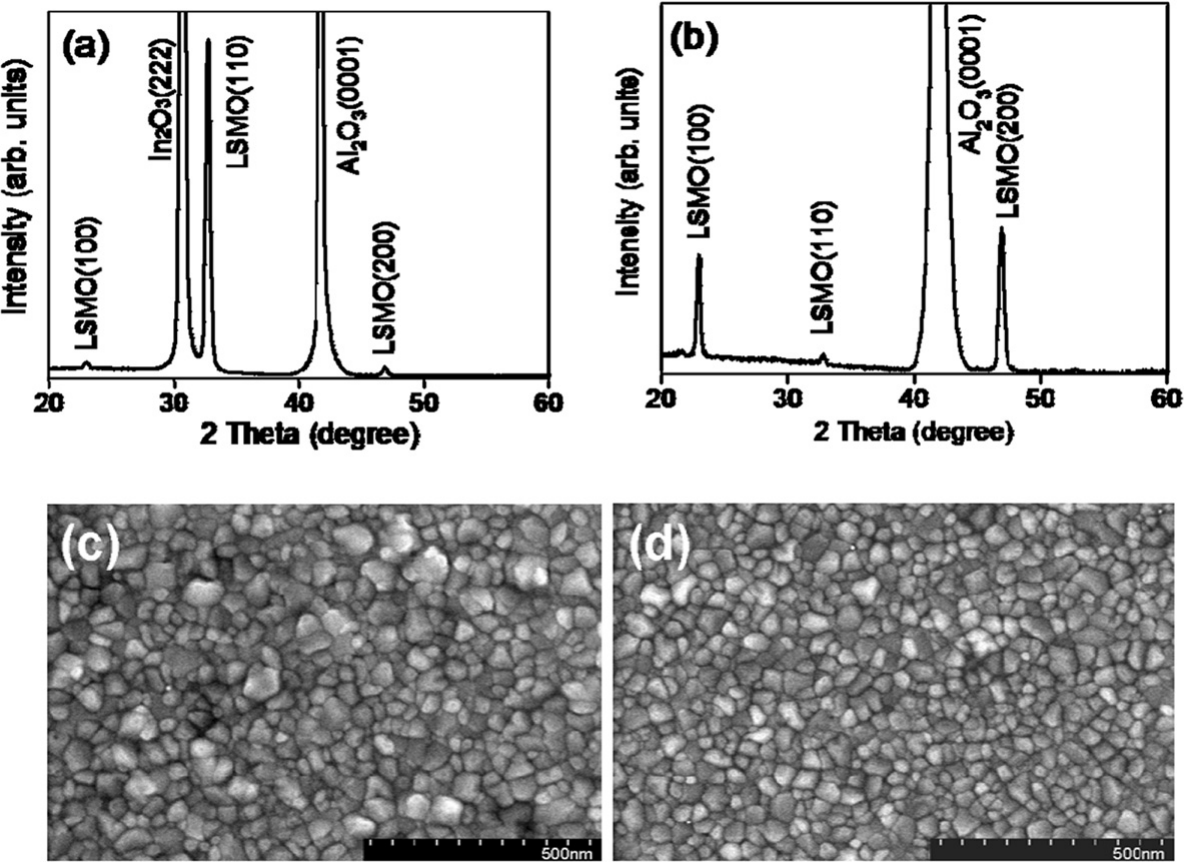
**XRD patterns and SEM images of LSMO nanolayer with and without In**_**2**_**O**_**3 **_**epitaxial buffering.** XRD patterns of LSMO nanolayer **(a)** with and **(b)** without In_2_O_3_ epitaxial buffering. SEM images of LSMO nanolayer **(c)** with and **(d)** without In_2_O_3_ epitaxial buffering.

Figure 1a,b shows the XRD patterns of the LSMO nanolayers grown on sapphire substrates with and without In_2_O_3_ epitaxial buffering, respectively. In addition to Bragg reflection from the In_2_O_3_ (222) and Al_2_O_3_ (0001) crystallographic planes, clear Bragg reflections of (100), (110), and (200) were present for the pseudo-cubic LSMO in the XRD measurement range. The XRD results show a highly (110)-oriented crystallographic feature of the LSMO nanolayer grown on the In_2_O_3_ (222) epitaxy. By contrast, a highly (*h*00)-oriented crystallographic feature was observed for the LSMO nanolayer grown on the bare sapphire substrate. The LSMO nanolayers with and without In_2_O_3_ epitaxial buffering are in a pseudocubic structure with a similar lattice constant of 0.387 nm. This is similar to the bulk value [4], demonstrating that no lattice distortion exists in the nanofilms. Interplanar spacing, corresponding to (110) LSMO plane (0.276 nm), is more similar to (222) plane of the In_2_O_3_ (0.292 nm) in comparison to the (100) LSMO plane. Moreover, a large lattice mismatch (approximately -13.2%) exists between In_2_O_3_ (222) and sapphire (0001) [13]. This information suggests that LSMO (110) growth on In_2_O_3_ (222) has a higher crystallographic compatibility degree during *in situ* crystal growth. Figure 1c,d shows the LSMO nanolayer SEM images with and without In_2_O_3_ epitaxial buffering, respectively. The grains are densely compacted, and no pores are found in the film surfaces. Furthermore, the grain size is more homogeneous for the LSMO nanolayers grown on the sapphire substrate. The LSMO grain sizes range from approximately 50 to 80 nm for the LSMO nanolayers on the sapphire substrate. The grains lying on the In_2_O_3_ epitaxially buffered sapphire substrate range from approximately 50 to 120 nm in size.

**Figure 2:**
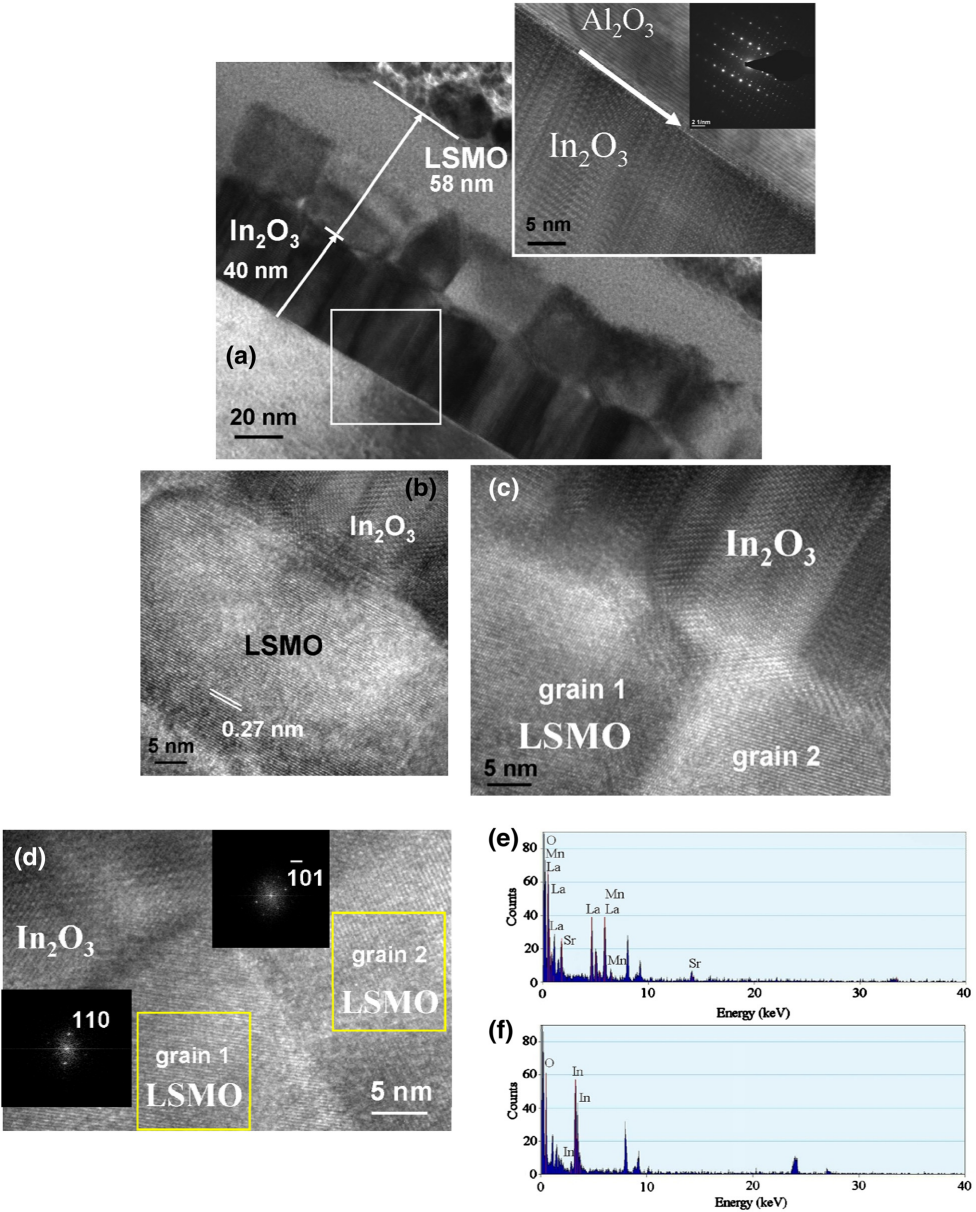
**TEM and HRTEM images and EDS spectra of LSMO nanolayer and In**_**2**_**O**_**3 **_**epitaxy. (a)** Low-magnification TEM image of the LSMO nanolayer with In_2_O_3_ epitaxial buffering on the sapphire substrate. The HRTEM image was taken from the interface of the In_2_O_3_ epitxay-sapphire substrate (white square region), and the inset shows the corresponding electron diffraction pattern at the heterointerface. **(b)** HRTEM image taken from the local single LSMO nanograin on the In_2_O_3_ epitaxy. **(c**, **d)** HRTEM images taken from the different local regions containing two neighboring LSMO nanograins on the In_2_O_3_ epitaxy. The corresponding FFT patterns taken from the different oriented LSMO nanograins are also shown in the insets of **(d)**. **(e)** EDS spectrum taken from the LSMO nanolayer. **(f)** EDS spectrum taken from the In_2_O_3_ epitaxy.

Figure 2a shows the cross-sectional TEM morphology of the LSMO nanolayer with In_2_O_3_ epitaxial buffering. The In_2_O_3_ epitaxy has approximately a 40-nm thickness and exhibits a columnar crystallite feature. The inset shows the In_2_O_3_ epitaxial high-resolution (HR) lattice fringes on the sapphire substrate. A clear interface was formed between the film and the substrate. The electron diffraction pattern taken from the interface of the In_2_O_3_ film and sapphire substrate also confirms that the In_2_O_3_ (222) epitaxial layer was grown on the *c*-axis-oriented sapphire substrate [11]. Moreover, a bilayer feature was observed on the LSMO nanolayer (Figure 2a). The total thickness of the LSMO nanolayer is approximately 58 nm, with a thinner 23-nm-thick homogeneous top sublayer, which is formed because of poor thin-film protection during the TEM sample preparation by focused ion beam milling. This may have caused a thermal effect and/or beam damage on the upper side of LSMO nanolayer. However, the lower side of the LSMO nanolayer maintained well crystalline granular features. The LSMO grains nucleated from the rugged surface of the columnar In_2_O_3_ epitaxy during thin-film growth. This caused the heterointerface between the LSMO nanolayer and In_2_O_3_ epitaxy to be rugged. Further investigation of the HR lattice fringes of one LSMO grain (Figure 2b) revealed that the interplanar d-spacing is approximately 0.276 nm in correspondence to the {110} lattice arrangement. A mechanism that matches the local domain epitaxy under a proper thin-film growth process demonstrated that it can form single-crystal LSMO grains with specific orientations [14]. Figure 2c,d shows the HR lattice fringes of the granular LSMO film taken from the different regions adjacent to the In_2_O_3_ epitaxy. A thin layer (approximately 2 nm in thickness marked with red boundaries) that serves as a transition boundary was formed between the LSMO nanograins and In_2_O_3_ epitaxy (Figure 2c). A similar crystallographic disorder, with an approximately 2-nm thickness, between the film and underlayer was shown in the perovskite LSMO and SrTiO_3_ epilayers grown on lattice mismatched materials [15]. This crystallographic disorder region is associated with a lattice strain relief between the film and the underlayer. The fast Fourier transformation (FFT) patterns in Figure 2d shows two misoriented nanograins. Depending on the relative rotation among the different grains during thin-film growth, the subgrain boundaries are formed among the nanograins. The TEM image shows that the subgrain boundaries on the nanometric scale combine the discrete-oriented crystallites to form a continuous LSMO nanolayer. Quantization of the spectrum in Figure 2e shows that the contents of La, Sr, Mn, and O are approximately 12.45, 7.85, 22.11, and 57.59 at %, respectively, for the LSMO thin layer. Therefore, approximately 38.7 at % of Sr dopant was achieved within the LSMO. Figure 2f exhibits that the element contents of the In_2_O_3_ layer are slightly oxygen deficient (the contents of In and O are approximately 46.19 and 53.81 at %, respectively). This is because the In_2_O_3_ epitaxy was grown under an oxygen-deficient atmosphere.

**Figure 3:**
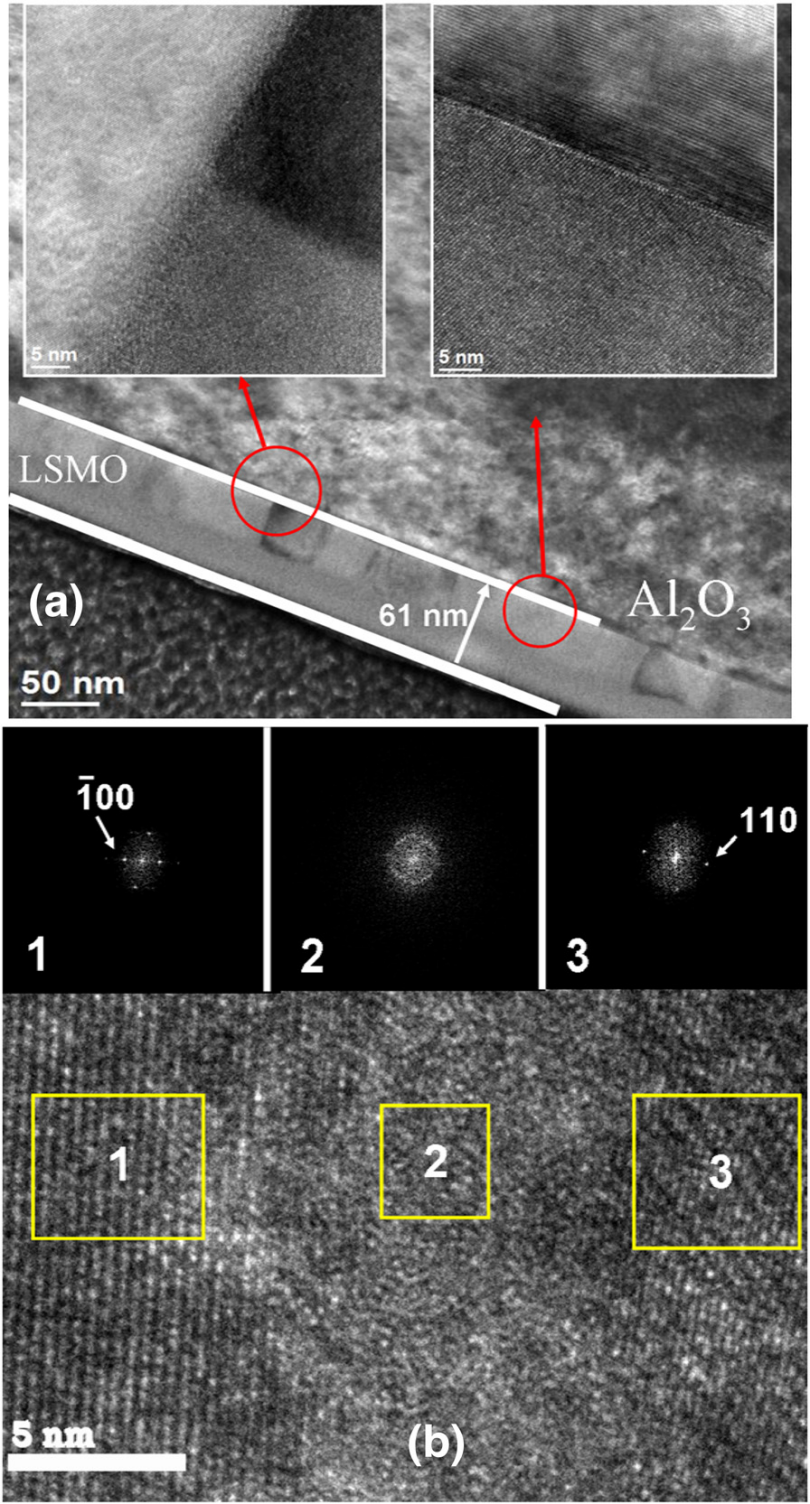
**Cross-sectional TEM morphology of the LSMO nanolayer, FFT patterns, and HR lattice fringes. (a)** Low-magnification TEM image of the LSMO nanolayer on the sapphire substrate. The insets show the HRTEM images of LSMO nanolayer on the sapphire with (right) and without (left) sharp interface. **(b)** HRTEM image taken from the local regions containing different oriented LSMO nanograins. The corresponding FFT patterns taken from regions 1, 2, and 3 are also shown.

Figure 3a shows the cross-sectional TEM morphology of the LSMO nanolayer grown on the bare sapphire substrate. A similarly damaged thin-layer was observed herein. Notably, granular LSMO layer contrast changes suggest that the film is composed of different LSMO crystallite orientations. Comparatively, the LSMO on the sapphire substrate experienced a relatively small degree of contrast changes, which cause the film structure to be more homogeneous than that on the In_2_O_3_ epitaxy. The insets show HR lattice fringes taken from different local regions at the interfaces between the LSMO nanograins and the sapphire substrate. Two types of heterointerface between the LSMO and substrate were presented. In the left inset, a thin (approximately 2 nm thick) transition layer formed at the heterointerface. By contrast, the right inset exhibits a high degree of interface coherency between the LSMO nanograin and substrate. The observation demonstrated that local single-crystal LSMO grains can be formed on the sapphire substrate with a sharp heterointerface during thin-film growth. The heterointerface between the LSMO nanolayer and the sapphire substrate is relatively flat and smooth in comparison to the one grown on the In_2_O_3_ epitaxy. This is believed to reduce the potential crystal defects at the heterointerface. Moreover, the FFT patterns and HR lattice fringes revealed that a thin disordered region was formed between the misoriented nanograins (Figure 3b).

**Figure 4:**
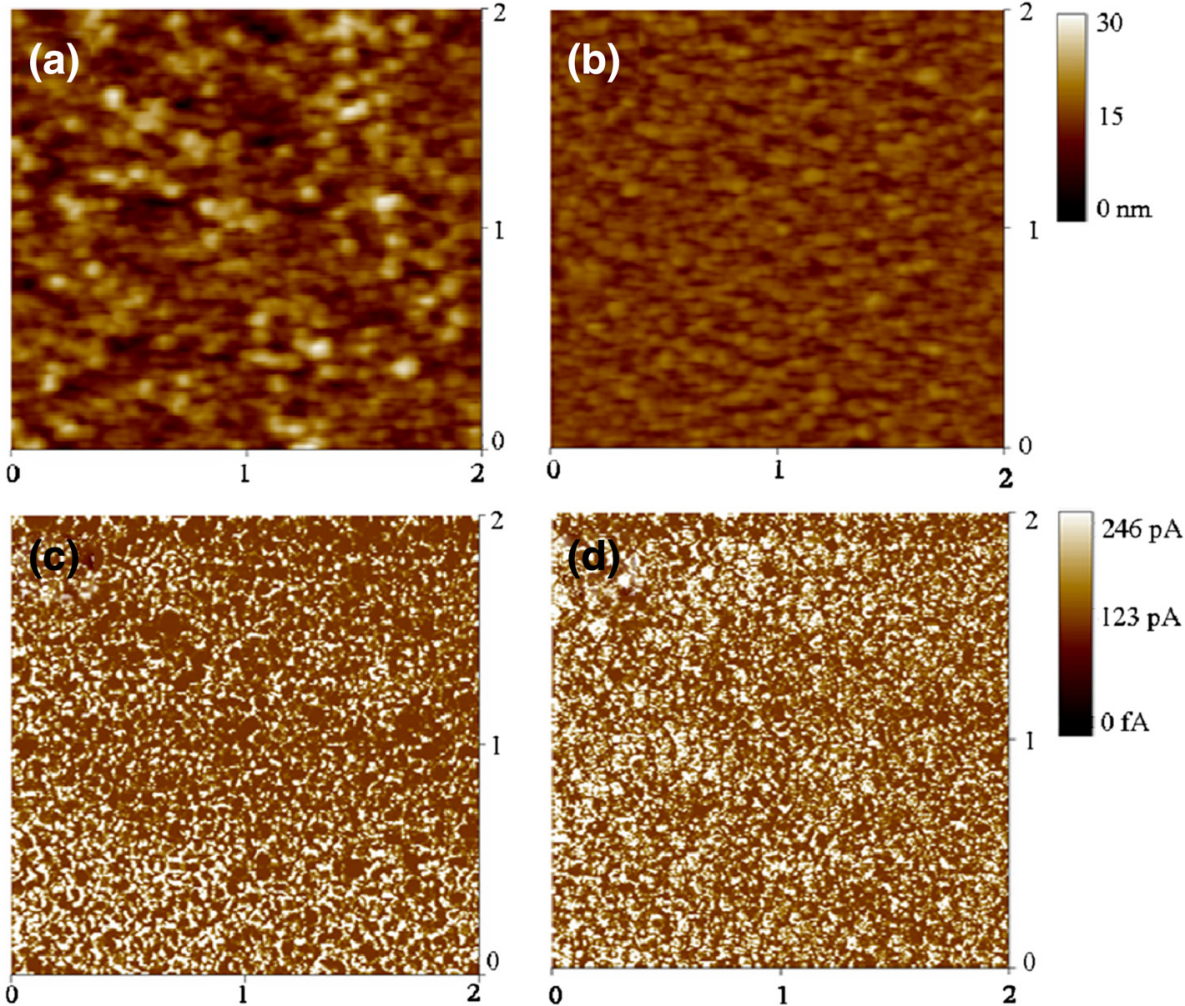
**AFM and CAFM images of the LSMO nanolayer.** AFM images of the LSMO nanolayer **(a)** with and **(b)** without In_2_O_3_ epitaxial buffering. CAFM images of the LSMO nanolayer **(c)** with and **(d)** without In_2_O_3_ epitaxial buffering.

Figure 4a,b shows the surface topography of LSMO nanolayers with and without In_2_O_3_ epitaxial buffering. Comparatively, with a root-mean-square (rms) roughness of 1.7 nm, the surface of the LSMO nanolayer grown on the bare sapphire substrate was smoother. The rms surface roughness of the film with In_2_O_3_ epitaxial buffering is 3.5 nm. As observed from the SEM images, the roughening of the LSMO nanolayer surface grown on the In_2_O_3_ epitaxy might be associated with its irregular grain sizes. Figure 4c,d shows the spatial distributions of currents at the micro- and/or nano-scale of the LSMO nanolayers with and without In_2_O_3_ epitaxy measured at a fixed applied bias during AFM scanning. The LSMO nanolayer current maps show that the dark regions only account for a remarkably small ratio over the area of interest, revealing that the LSMO nanolayer surfaces remain a conductive characteristic under 0.05V. In comparison, the LSMO nanolayer without In_2_O_3_ epitaxial buffering has a homogeneously spatial distribution of current spots over the measured area. The current mean statistic value distributed over the measured area is 30.3 and 38.8 pA for the LSMO nanolayers with and without In_2_O_3_ epitaxial buffering, respectively. The LSMO nanolayer with In_2_O_3_ epitaxial buffering is slightly more resistant than the film without buffering.

**Figure 5:**
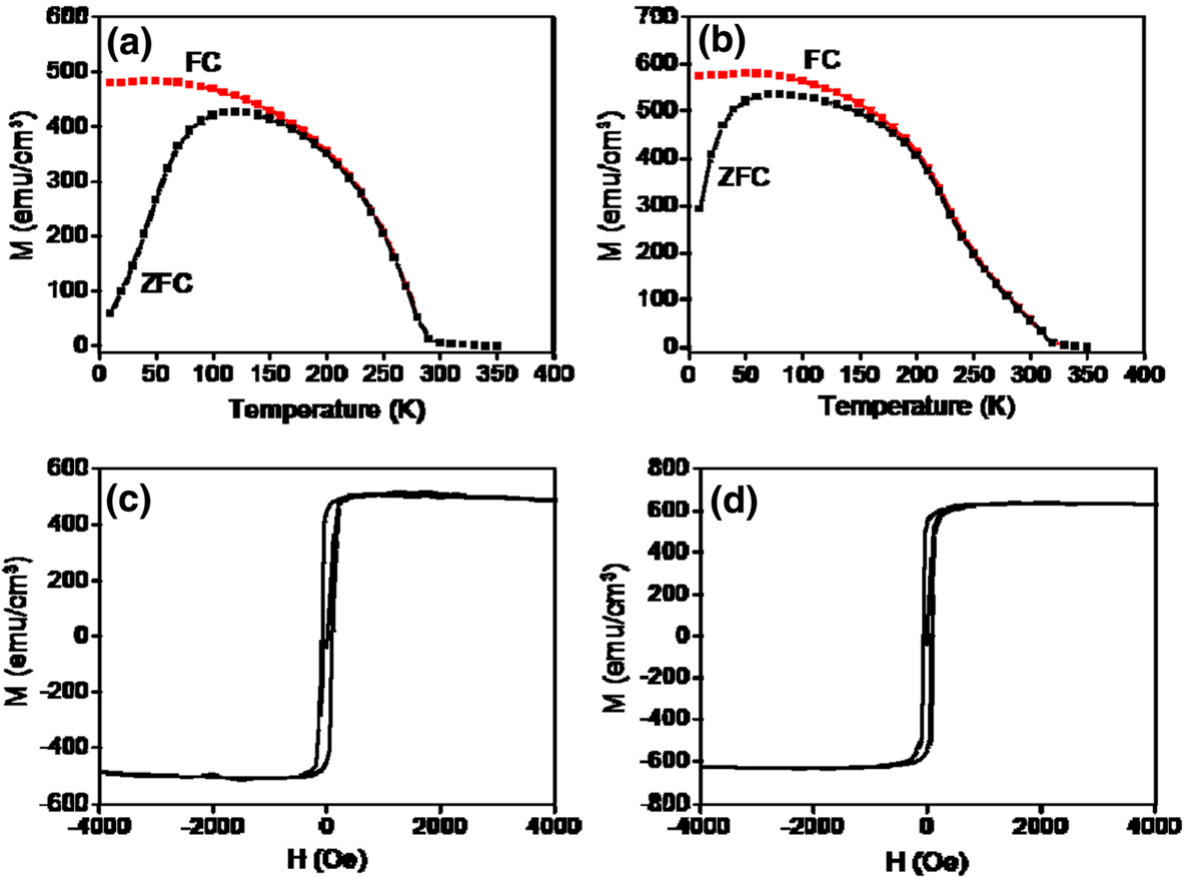
**FC and ZFC *M*-*T *curves.** Field-cooled and zero-field-cooled *M*-*T* curves of the LSMO nanolayer **(a)** with and **(b)** without In_2_O_3_ epitaxial buffering. *M*-*H* curve of the LSMO nanolayer **(c)** with and **(d)** without In_2_O_3_ epitaxial buffering.

Figure 5a,b shows the magnetization vs. temperature curves (*M*-*T*) for the zero-field-cooled (ZFC) and field-cooled (FC) samples. The applied magnetic field was 1,000 Oe during the *M*-*T* measurements. The *M*-*T* curves demonstrated that the LSMO nanolayers have a sharp ferromagnetic to paramagnetic transition. Comparatively, a higher magnetization and Curie temperature was observed for the LSMO nanolayers without In_2_O_3_ epitaxial buffering. The Curie temperatures of the LSMO nanolayers with and without In_2_O_3_ epitaxial buffering were 290 and 323K, respectively. A higher ferromagnetic ordering degree causes the LSMO films to have a higher saturation magnetization value and Curie temperature [16]. This reveals that more structural inhomogeneities in the LSMO nanolayer with In_2_O_3_ epitaxial buffering caused the double-exchange mechanism to have a greater depression degree [17]. Moreover, the higher moment in manganite thin films was attributed to a lower resistivity of the film [18]. This is in agreement with the CAFM measurements that convey that the LSMO nanolayer with In_2_O_3_ epitaxial buffering is slightly more resistant than the film without buffering. There is a large difference in the ZFC and FC curves’ low temperature range. ZFC curves display a broad summit peak. A larger difference in magnetization between the ZFC and FC curves in the low temperature region was observed for the LSMO nanolayer with In_2_O_3_ epitaxial buffering, which conveyed that randomly oriented magnetic domains are more difficult to align in the film. The subgrain boundaries among the LSMO nanograins, rough film surfaces, and interfaces caused an existence of disordered spins in the LSMO nanolayer. These disordered spins might play an important role in separating the magnetically ordered regions in the LSMO nanolayer [19]. This caused the marked cluster glass state in the film. Figure 5c,d shows the magnetization-field (*M*-*H*) hysteresis curves at 50 K for LSMO nanolayers with and without In_2_O_3_ epitaxial buffering. The field was applied parallel to the substrates. The respective in-plane saturated magnetization value was approximately 500 and 625 emu/cm^3^ for the LSMO nanolayers with and without In_2_O_3_ epitaxial buffering, respectively. The LSMO nanolayers with and without In_2_O_3_ epitaxial buffering have coercive fields that are 90 and 72 Oe, respectively. The crystal imperfections, such as surface roughness, subgrain boundary, and heterointerface, play important roles in determining the coercivity [7]. Several results conveyed that the surface roughness provides an extra hindrance to the magnetization reversal and induces an increase in coercivity accordingly [20]. Moreover, a greater degree of structural inhomogeneities (rugged heterointerfaces and subgrain boundaries) in the LSMO nanolayer with In_2_O_3_ epitaxial buffering act as domain-wall pinning centers [17]. The relatively low coercivity is attributed to the high quality, low defect density of the LSMO nanolayer without buffering. The structural analyses support the observed *M*-*H* results.

## Conclusions

In summary, 60-nm-thick LSMO nanolayers were grown on sapphire substrates with and without In_2_O_3_ (222) epitaxial buffering. The LSMO experienced improved (110) preferred crystal growth via In_2_O_3_ (222) epitaxial buffering. Comparatively, the surface grain size is more homogeneous for the LSMO nanolayer grown on the sapphire substrate. The rugged surface of the In_2_O_3_ epitaxial underlayer further incurred rougher surface morphology of the LSMO nanofilm. The columnar crystallite feature of the In_2_O_3_ epitaxial underlayer caused a relatively smaller lateral domain size of the manganite ultra-thin layer on it. Moreover, In_2_O_3_ epitaxial buffering resulted in rugged heterointerfaces between the LSMO nanolayer and In_2_O_3_ epitaxy. These factors contributed to a higher content of subgrain boundaries and incoherent interfaces on a nanometric scale in the LSMO nanofilm via In_2_O_3_ epitaxial buffering. These disordered regions caused disordered spins to exist in the LSMO nanolayer. Therefore, lower saturation magnetization value and Curie temperature, and higher coercivity and resistivity are found in the highly (110)-textured LSMO nanolayer.

## Competing interest

The authors declare that they have no competing interests.

## Authors’ contributions

YCL designed the experiments and drafted the manuscript. HZ carried out the thin-film preparation and material analyses. WKL analyzed the AFM and CAFM data. All authors read and approved the final manuscript.

## Authors’ information

YCL is a professor of the Institute of Materials Engineering at National Taiwan Ocean University (Taiwan). HZ received his Masters degree in Materials Engineering at National Taiwan Ocean University (Taiwan) in 2013. WKL is a graduate student of the Institute of Materials Engineering at National Taiwan Ocean University (Taiwan).
